# Prioritising and mapping barriers to achieve equitable surgical care in South Africa: a multi-disciplinary stakeholder workshop

**DOI:** 10.1080/16549716.2022.2067395

**Published:** 2022-06-22

**Authors:** Tamlyn Mac Quene, Luné Smith, Maria Lisa Odland, Susan Levine, Lucia D’Ambruoso, Justine Davies, Kathryn Chu

**Affiliations:** a Centre for Global Surgery, Department of Global Health, Faculty of Medicine and Health Sciences, Stellenbosch University, Cape Town, South Africa; b Faculty of Medicine and Health Sciences, Stellenbosch University, Cape Town, South Africa; c Institute for Applied Health Research, University of Birmingham, Birmingham, UK; d Department of Anthropology, Humanities Faculty, University of Cape Town, Cape Town, South Africa; e Aberdeen Centre for Health Data Science, Institute of Applied Health Sciences, School of Medicine, Medical Sciences and Nutrition, University of Aberdeen, Aberdeen, Scotland, UK; f Faculty of Health Sciences, Medical Research Council/Wits University Rural Public Health and Health Transitions Research Unit, University of Witwatersrand, Johannesburg, South Africa

**Keywords:** Barriers to care, priority setting, surgery, health systems, South Africa

## Abstract

**Background:**

Surgical healthcare in South Africa is inequitable with a considerable lack of resources in the public health sector. Identifying barriers to care and creating research priorities to mitigate these barriers can contribute to strategic interventions to improve equitable access to quality surgical care.

**Objective:**

To use the Four Delays Framework to map barriers to surgical care and identify priorities to achieve equitable and timely access to quality surgical care in South Africa.

**Methods:**

A multi-disciplinary stakeholder workshop was held in Cape Town, South Africa in January 2020. A Four Delays Framework (delays in seeking care, reaching care, receiving care, and remaining in care) was used to identify barriers that occur at each delay and the top 10 priorities for intervention. Barriers were categorised into overarching themes and schematically mapped.

**Results:**

Thirty-four stakeholders including health service users, health service providers, and community members participated in this exercise. In total, 34 barriers were identified with 73 connections to various delays. Specifically, 14 barriers were related to delays in seeking care, 11 were related to delays in reaching care, 20 were related to delays in receiving care, and 28 were related to delays in remaining in care. The highest priority barriers across the delays were *Lack of service provider’s knowledge, training and experience*, and *Limited surgical outreach*. The barrier *Lack of decentralised services* was related to all four delays. Barriers were interconnected and potentially reinforcing.

**Conclusions:**

This workshop is the first of its kind to generate evidence on the delays to surgical care in South Africa. Mapping crucial interconnected, potentially reinforcing barriers, and priority interventions demonstrated how a multifaceted approach may be required to address delays to access. Further research focused on the identified priorities will contribute to efforts to promote equitable access to quality surgical care in South Africa.

## Background

Deaths due to surgically treatable conditions are higher than those from HIV/AIDS, tuberculosis, and malaria combined in low- and middle- income countries (LMICs) [1,2]. Access to timely and quality surgical care saves lives but several barriers impede this, particularly in LMICs, where nine out of ten people cannot access basic surgical care and only 3.5% of approximately 234 million major surgical operations performed worldwide are undertaken in low-resource countries [1,3]. Low operative volumes are exacerbated by the shortage of surgical providers, infrastructure, and resources pertinent to delays to quality surgical provision in these settings [4]. Disparities in access are further affected by many interconnected barriers that include sociocultural factors like stigmatisation around surgery, financial factors including direct and indirect costs of care and many health system related factors [5].

The Three Delays Framework [6,7] was developed to understand the multiplicity of barriers driving avoidable maternal deaths and is becoming increasingly used to examine barriers to access to care for other conditions, including surgical care, trauma care, and other time-critical conditions [8–11]. Barriers are experienced at three delay stages: 1) seeking; 2) reaching; and 3) receiving quality health care [1]. Various studies from LMICs have used the Three Delays Framework to show that people experience barriers at multiple stages in the care pathway resulting in delays to accessing surgical care [12–14]. For example, a study in Somaliland reported delays for children receiving or seeking surgical care were most prominent due to barriers such as no perceived need or seeking traditional health care [15]. Similarly, a study conducted in Ghana reported that the lack of appropriate care from the nearest health facility was the biggest barrier to receiving and seeking care [16]. Moreover, a recent systematic review on trauma care found 111 studies assess these delays in LMICs with most focusing on the delay to receiving care and only 2.7% of studies assessed all delays [14]. Recently, the Three Delays Framework has been expanded to the Four Delays Framework by adding *remaining in care* to better understand retention issues within the health system as another delay to accessing care [11,17]. Many studies have identified barriers to access to care, but these have often focussed on one or few delay stages or involved limited stakeholders. Whereas to address delays in access to quality care for surgery in LMICs effectively requires a holistic understanding of barriers to access from multiple stakeholder viewpoints.

South Africa, in particular, has a high burden of surgical conditions such as traumatic injuries, cancer, and other complications of non-communicable and communicable diseases [18,19]. South Africa is one of the most unequal countries in the world, with a Gini coefficient of 0.63 [20]. This inequity is reflected in access to healthcare, despite a national commitment to Universal Health Coverage [21,22]. Currently, the under-resourced public health sector serves 84% of the population compared to 16% being served by the private health sector [23,24]. Resource constraints in the public health system compounded by socio-economic challenges, particularly those experienced by marginalised groups, can impact access to surgical care [25,26]. Ensuring access to quality surgical care requires the availability and interconnectivity of multiple facets of the health system. Therefore, in order to improve surgical care services in South Africa, it is necessary to understand the totality of the barriers that healthcare service users experience in accessing care and how these barriers are interconnected [27]. Thus, the aim of this workshop was to use the Four Delays Framework to map barriers to surgical care and identify which were priorities to achieve equitable and timely access to quality surgical care in South Africa.

## Methods

### Study setting

South Africa is an upper-middle-income country with a population of approximately 60 million people [24]. The health system is comprised of a private and public (government) sector. Public health facilities are located and managed by provincial health departments, which provide health services through an integrated system of community health clinics and hospitals. Public hospitals are further divided into district, secondary, and tertiary hospitals with increasing speciality and surgical services offered at higher-level facilities.

A multi-disciplinary stakeholder workshop was held in Cape Town, South Africa in January 2020, whereby stakeholders identified and prioritised barriers to accessing surgical care in the South African public health sector.

### Stakeholders

Invited stakeholders included health service users, health service providers, and community members from rural and urban settings within the Western and Eastern Cape provinces. Health service providers and community members were invited by telephone call and email respectively. Health service users were invited by a telephone call from former health service providers. Health service users were defined as people who had previously received surgical care within the public health sector. Health service providers included emergency medicine physicians, surgeons, anaesthesiologists, obstetricians, primary care physicians, public health specialists, nurses, and medical students. Community representatives included community health committee members from two local health districts, Khayelitsha and Klipfontein. Facilitators with extensive experience in leading workshops on exploring barriers to healthcare in LMICs facilitated the sessions. The facilitators did not work directly in the South African surgical healthcare system so they could be as objective as possible. Two of the facilitators were social scientists and two were clinicians and global surgery specialists.

Stakeholders were recruited purposively from amongst the authors’ networks, to ensure a balanced representation of age, sex, ethnic backgrounds, and geographical location of living or practice [28]. Public sector service providers identified former service users who were willing to speak on their experiences within the surgical health system. Service users with varying previous surgical conditions (covering traumatic injuries, cancer, and infectious conditions) were recruited.

### Identifying and prioritizing barriers

**Figure 1.**
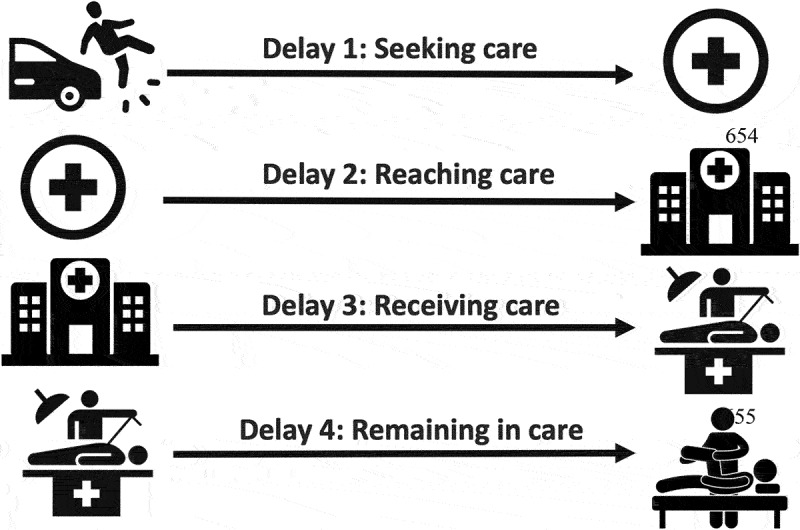
The Four Delays Framework.

After a short introduction to the Four Delays Framework, stakeholders were divided into four groups (8–10 stakeholders per group) based on their roles as service users, service providers, or community members (Figure 1). Stakeholders were placed into specific delay groups based on expertise and experience. Service users were placed into Group 1: Seeking care and Group 4: Remaining in care. Service providers were placed into Group 2: Reaching care and Group 3: Receiving care. Community members were spread throughout the groups equally. Service users and community members were separated from service providers as much as possible in order to mitigate the perceived power imbalances between service user and service provider, which might have limit expression of opinions.

The workshop was divided into three 45-minute sessions each followed by a plenary discussion (30 minutes to 1 hour) and managed by the lead facilitator. Facilitators encouraged all stakeholders to speak freely. During the first session, each group identified and discussed barriers which acted in their assigned delay through brainstorming and consensus. All barriers were then presented to all stakeholders during the plenary discussion. Where identified barriers acted equally at different delays, they were assigned to each of these delays. During the second session, each group listed the identified barriers by priority for intervention. Group members were asked to prioritise barriers based on considerations of impact (e.g. number of lives saved) and cost. However, this criterion was not fixed, and stakeholders were able to prioritise other barriers that did not fulfil these criteria, but nevertheless were considered by their group as a priority. The top four prioritised barriers from each group and the rationale for this prioritisation were presented to all the stakeholders. At the third plenary session, stakeholders ranked the top 10 intervention priorities overall through consensus building. Consensus was reached when all stakeholders agreed to the relevance and priority of each barrier after several rounds of discussion followed by voting.

### Mapping barriers to care

Barriers, their related delays, and their priority for intervention were captured by the facilitators. The writing group, using an iterative process, refined the barriers to ensure that duplicates or overlapping barriers were merged or removed. All barriers to surgical care were categorised into the Four Delays Framework and overarching themes. Overarching themes were defined as individual, societal, and financial factors specific to accessing the public health sector, non-healthcare related infrastructure, and the health system. These themes were derived from health system frameworks previously used in another Four Delays study and were based upon Atunet al’s consideration of a health system as including wider societal factors as well as health service factors [11,29,30]. Barriers, their delays, their interconnections with other barriers, and overarching themes were then tabulated.

The barriers were schematically mapped into a Four Delays Framework using Stella Architect Trial Version 1.9.5 software. Mapping was done by spatially relating each barrier to its overarching themes and delays. Once all barriers were mapped, their interconnection was illustrated with arrows. Arrowheads were not related to the direction of connectedness or influence.

### Ethical considerations

This was a priority setting stakeholder workshop which did not report any personal information, or utilise any individual quotes, or other identifying material. Potential for social harm or risk towards stakeholders included feelings of being stereotyped or emotional harm recalling barriers to surgical care. There were no immediate benefits for stakeholders however, the findings of the workshop could improve the healthcare system where they work or receive care. Stakeholder consent was granted for use of the material generated for publication.

## Results

In total, there were 34 South African-based stakeholders. Service providers included: surgeons (2), obstetricians (2), anaesthesiologist (1), family physicians (5), emergency medicine physician (1), medical students (4), nurse (1), and clinical manager (1). There were 10 service users and seven community representatives (Supplementary Table 1).

Initially, 59 barriers were identified by the stakeholders. After further refinement and removal of 24 overlapping barriers, 34 barriers remained. Of the 34 barriers identified, 14 barriers were related to the delay in seeking care, 11 to the delay in reaching care, 20 to the delay in receiving care, and 28 to the delay in remaining in care (Table 1). Overlapping barriers were merged. For example, *Lack of community support*, and *Lack of practical support from family and community* were merged to *Lack of practical support from community. Complex and disjointed referral system*, and *Lack of functional referral system* were merged to *Complex and disjointed referral system. Lack of social work and home-based support* was renamed *Lack of home-based services. Lack of funding* was renamed *Lack of funding in the public healthcare system. Cost of accessing private healthcare* was removed because it was not relevant to accessing the public healthcare system.

**Table 1.** Barriers to surgical care and their related delays

Barrier	Delay(s)*
Lack of decentralised services	1, 2, 3, 4
Family responsibilities	1, 2, 4
Lack of social support	1, 2, 4
Experienced lack of staff empathy	1, 3, 4
Language barrier	1, 3, 4
Systemic inequities	1, 3, 4
Lack of service user’s surgical healthcare knowledge	1, 4
Lack of surgical health education	1, 4
Procrastination	1, 4
Religion/preference for alternative practitioners	1, 4
Stigma of disease	1, 4
Fear of the individual consequences of surgical care	1
Previous bad experiences	1
Wariness of healthcare system	1
Cancellation of appointments and procedures	2, 3, 4
Lack of continuity of care	2, 3, 4
Lack of funding of public healthcare system	2, 3, 4
Poor communication between service providers at different levels	2, 3, 4
Complex and disjointed referral system	2, 4
Poor road infrastructure	2, 4
Delay of diagnosis	2, 3
Problems with cost, time, safety, distance and comfort of transport	2
Difficulty with navigating the facility	3, 4
Lack of adequate service user records	3, 4
Lack of and poor maintenance of equipment	3, 4
Lack of management, referral and retention in care guidelines	3, 4
Lack of service provider’s knowledge, training and experience	3, 4
Lack of supervision for junior service providers	3, 4
Large burden of disease	3, 4
Limited surgical outreach	3, 4
Long waiting times	3, 4
Lower prioritisation of certain surgical conditions	3, 4
Limited theatre time	3
Lack of home based services	4

*Delay 1: seeking care, Delay 2: reaching care, Delay 3: receiving care, Delay 4: remaining in care.

The top 10 priority barriers to accessing surgical care in South Africa are shown in Table 2. The top three barriers were *Lack of service provider’s knowledge, training and experience*, and *Limited surgical outreach*, and *Lack of and poor maintenance of equipment*.

**Table 2.** Priority barriers to accessing surgical care in South Africa

Priority	Barrier	Votes
1	Lack of service provider’s knowledge, training and experience	12
2	Limited surgical outreach	11
3	Lack of and poor maintenance of equipment	9
4	Lack of surgical health education	8
5	Lack of decentralised services	8
6	Lack of continuity of care	7
7	Long waiting times	7
8	Complex and disjointed referral system	7
9	Problems with cost, time, safety, distance and comfort of transport	6
10	Lack of social support	5

### Barrier mapping

**Figure 2.**
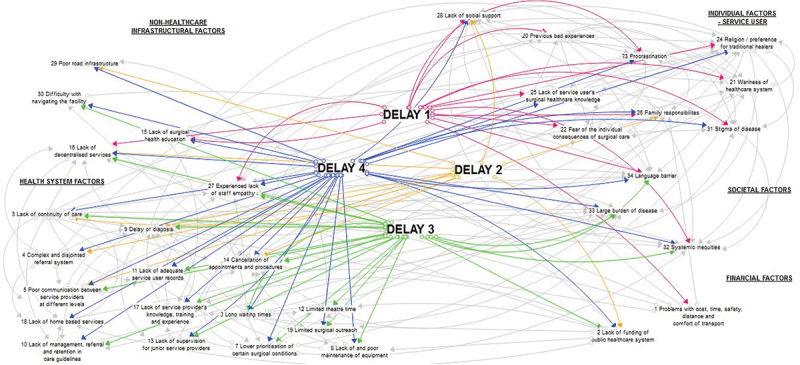
Map showing barriers, their interconnection, and their relationship with the overarching themes and delays.

All interconnected barriers, delays, and overarching themes are shown in Supplemental Table 2. The most prominent theme was health system factors. Barriers were connected across a complex network of overarching themes and delays (Figure 2). The spatial relationship between barriers and themes was illustrated by *Religion/preference for alternative practitioners* which was mapped halfway between individual factors and societal factors as it was equally relevant to both themes (Figure 2). Supplemental Figures 1–4 map each delay and its barriers.

The visual representation of the mapped barriers shows the interconnections between the barriers and delays. *Delay of diagnosis* connects to 19 other barriers. One of the highest prioritised barriers, *Lack of service provider’s knowledge, training, and experience*, connects to six other barriers, such as *Lack of funding of the public healthcare system* (delays in reaching, receiving, and remaining in care), *Long waiting times* (delays in receiving and remaining in care) and *Experienced lack of staff empathy* (delays in seeking, receiving, and remaining in care).

## Discussion

This multi-disciplinary stakeholder workshop brought together service users, service providers, and community members. Together, stakeholders identified several interconnected barriers which impact equitable and timely access to quality surgical care in South Africa.

Using the Four Delays Framework, we illustrated 34 barriers and their overarching themes that can lead to delays in accessing quality surgical care when needed. The barrier *Lack of decentralised services* related to all four delays, ranked in the top 10 priorities for intervention, and linked to five other health system-related barriers including *Lack of continuity of care, Complex and disjointed referral system, Long waiting times, Delay of diagnosis*, and *Lack of home-based services*. The 2015 World Health Assembly Declaration 68.15 which stated essential and emergency surgical care as a key component to Universal Health Coverage, placed the district hospital as the backbone of achieving quality surgical access for all [31]. The lack of district hospital surgical capacity in South Africa is a critical limitation to improving surgical capacity and mitigating this barrier, and should be one of the critical focuses of any national or provincial surgical improvement strategy [32,33].

Several barriers in the top 10 priority list were related to delays in receiving care (delay three) and remaining in care (delay four), highlighting the importance of facility-level access. Other studies that utilised the Delays Framework have also found barriers to receiving care as an important delay in accessing surgical care [15,29,34,35]. Additionally, a study examining avoidable mortality after trauma in rural South Africa found that delays in receiving quality care were most common [10]. However, another study done in rural South Africa for time-critical conditions and non-communicable diseases highlighted seeking care as the dominant cause of delay [9], suggesting that even within one country, geographical location and disease type may influence barriers to access to care.

The highest priority for intervention was *Lack of service provider’s knowledge, training and experience* which related to delays in receiving and remaining in care. Other studies have demonstrated that the shortage of appropriate human resources is associated with delays to service provision at health facilities in LMICs [8,36,37]. Moreover, recent studies in South Africa have demonstrated that the lack of adequately trained service providers contributed to disparities between the care received in the public and private health sector, further compounding inequitable access to surgical care [38–40]. The upskilling and training of service providers is needed to improve surgical capacity at public health facilities, and in turn, access to quality surgical care. Methods of addressing the lack of service providers’ knowledge and experience may include in-service training, task-shifting [41], and virtual consultations and mentoring as a short-term solution to the lack of surgical expertise [4].

The second highest priority was *Limited surgical outreach*. A surgical outreach program in rural South Africa demonstrated that successful delivery of point of care can be achieved by taking surgical expertise to district hospitals [42]. While the program did not result in increased surgical capacity at the facilities, it may have contributed to timely identification and referral of conditions from rural areas to appropriate health facilities [42]. In addition, a surgical outreach initiative in Uganda was associated with increased surgical outputs [43]. The effects of such surgical outreach programs in other provinces in South Africa need to be investigated.

*Lack of and poor maintenance of equipment* was the third barrier prioritised for intervention. Several previous studies have shown that facilities in LMICs have a shortage of the necessary surgical equipment and infrastructure to provide timely and quality surgical care [4,44,45]. In particular, more complex elective operations are often delayed due to a shortage of operating theatre time and the lack of essential equipment [39,45]. Ensuring the availability of functioning equipment is essential to decrease long waiting times and reduce associated mortality and morbidity [44].

While our results were generated in South Africa, our broad findings could help understand delays to surgical care in other settings. In general, our results show multiple barriers with many spanning more than one delay stage or overarching theme. The illustrated complexity, interactivity, and likely reinforcing nature of barriers to access of surgical care is likely to be seen in all other healthcare settings regardless of whether individual barriers are shared. Our results reinforce the need to understand the health system as a complex and adaptive system when considering improving surgical services, wherever those improvements are being considered.

Our workshop had limitations. Firstly, South Africa has a heterogeneous population and our results could be limited by sampling. We tried to mitigate this by recruiting a diverse group of stakeholders through purposive sampling based on background, ethnicity, rurality, and type of surgical expertise/condition. The 34 stakeholders included were from only two of nine South African provinces and our results on specific barriers might not be generalisable. Nevertheless, our findings of the number and interconnectedness of the barriers and their interactions across multiple delay stages and thematic areas are highly likely to generalise to other settings. Secondly, workshop stakeholders may not have reported all known barriers for fear of stigmatization or reprisals. Thirdly, government health officials and policymakers were also not included as stakeholders and therefore their perspective on the pragmatic implementation of prioritised barriers for intervention was lacking. Lastly, the refinement of the barriers for the schematic map visualization was undertaken by the writing group only (LS, MLO, TM, KC, and JD) and no feedback was obtained from the rest of the stakeholders. Despite these limitations, the strength of our findings lies in the diversity of stakeholders. While other prioritisation exercises have only included service providers [11], our stakeholder workshop – which included former surgical patients and community representatives – minimised stakeholder bias and legitimises our results.

## Conclusion

To our knowledge, this multi-disciplinary stakeholder workshop is the first of its kind to generate evidence on the delays to surgical care in South Africa. Critical interconnected barriers and intervention priorities were identified and mapped. This allowed for a targeted approach to be adopted that may have a cascading impact on access to quality surgical care. Practical and policy considerations need to be considered when designing interventions to improve access to surgical care. Availability of standardised data on surgical indicators that are comparable, scale-up of surgical capacity and services through decentralisation and the increase of surgical providers are needed. Evidenced-based research to increase political commitment and generate national government buy-in to increase the priority and inclusion of surgical care into national health plans as well as financial support are crucial. Overall, the findings from this workshop serve to support further research around designing interventions to strengthen access to surgical care and advance surgical equity for the South African healthcare system.

## Supplementary Material

Supplemental Material

## Data Availability

The datasets generated and/or analysed during the current study are not publicly available.

## Paper context

Surgical care is inequitable in South Africa and delays to accessing care could be contributing factors. This stakeholder workshop is the first of its kind to generate evidence on the delays to surgical care and highlights several crucial interconnected barriers that contribute to the discourse around access to safe, timely and quality surgical care in the South African context. Further research around designing interventions to strengthen equitable access to surgical care is needed.
