# Supplementary material for: Prioritising and mapping barriers to achieve equitable surgical care in South Africa: a multi-disciplinary stakeholder workshop
Source: Glob Health Action. 2022 Jun 22;15(1):2067395. doi: 10.1080/16549716.2022.2067395 (PMC9225684; doi:10.1080/16549716.2022.2067395)
Supplement: Supplemental Material [file ZGHA_A_2067395_SM5904.docx]

Supplementary Table 1: Role and expertise of the workshop stakeholders

|  | **Role** | **Surgical Condition/Expertise** | **Assigned Delay** |
| --- | --- | --- | --- |
| 1 | Service user | Breast cancer | 1 |
| 2 | Service user | Breast cancer | 4 |
| 3 | Service user | Colorectal cancer | 1 |
| 4 | Service user | Colorectal disease | 1 |
| 5 | Service user | Colorectal disease | 4 |
| 6 | Service user | Colorectal disease | 4 |
| 7 | Service user | Colorectal disease | 4 |
| 8 | Service user | Inflammatory bowel disease | 1 |
| 9 | Service user | Traumatic Injury | 1 |
| 10 | Service user | Traumatic Injury | 4 |
| 11 | Community member | Community health forum 1 | 1 |
| 12 | Community member | Community health forum 2 | 1 |
| 13 | Community member | Community health forum 1 | 2 |
| 14 | Community member | Community health forum 2 | 2 |
| 15 | Community member | Community health forum 1 | 3 |
| 16 | Community member | Community health forum 1 | 4 |
| 17 | Community member | Community health forum 2 | 4 |
| 18 | Service provider | Anaesthesiologist | 3 |
| 19 | Service provider | Breast surgeon | 2 |
| 20 | Service provider | Colorectal surgeon | 3 |
| 21 | Service provider | Emergency medicine physician | 2 |
| 22 | Service provider | Family physician | 2 |
| 23 | Service provider | Family physician | 2 |
| 24 | Service provider | Family physician | 2 |
| 25 | Service provider | Family physician/Public health specialist | 3 |
| 26 | Service provider | Family physician/rural doctor | 3 |
| 27 | Service provider | Medical student | 1 |
| 28 | Service provider | Medical student | 2 |
| 29 | Service provider | Medical student | 3 |
| 30 | Service provider | Medical student | 4 |
| 31 | Service provider | Obstetrician/Gynaecologist | 2 |
| 32 | Service provider | Obstetrician | 3 |
| 33 | Service provider | Operating Theatre manager | 2 |
| 34 | Service provider | Surgical outpatient nurse | 3 |

Supplementary Table 2. Barriers, connected barriers and their delays.

| **Barrier Number** | **Barrier** | **Explanation** | **Barrier Connections** | **Delay(s)*** | **Dominant Overarching Theme** |
| --- | --- | --- | --- | --- | --- |
| 1 | Problems with cost, time, safety, distance and comfort of transport |  | 9, 16, 26, 28, 29 | 2 | Financial factors |
| 2 | Lack of funding of public healthcare system |  | 8, 12, 15, 16, 17, 18, 19 | 2, 3, 4 | Financial factors |
| 3 | Lack of continuity of care | Service provider's provision of consistent care through collaboration with other service providers. | 5, 7, 9, 10, 11, 14, 16, 18, 21, 24, 27, 32, 33 | 2, 3, 4 | Health system factors |
| 4 | Complex and disjointed referral system |  | 5, 9, 16, 18, 24 | 2, 4 | Health system factors |
| 5 | Poor communication between service providers at different levels |  | 3, 4, 9, 11, 34 | 2, 3, 4 | Health system factors |
| 6 | Long waiting times | Service users waiting for a consultation or waiting to be seen at the facility. | 7, 8, 9, 10, 12, 14, 16, 17, 19, 21, 24, 33 | 3, 4 | Health system factors |
| 7 | Lower prioritisation of certain surgical conditions | Surgical care given to emergency conditions first, delaying/preventing care of chronic conditions | 3, 6, 9, 12, 14, 21, 32, 33 | 3, 4 | Health system factors |
| 8 | Lack of and poor maintenance of equipment |  | 2, 6, 9, 12, 14 | 3, 4 | Health system factors |
| 9 | Delay of diagnosis |  | 1, 3, 4, 5, 6, 7, 8, 10, 13, 14, 16, 17, 20, 21, 23, 24, 32, 33, 34 | 2, 3 | Health system factors |
| 10 | Lack of management, referral and retention in care guidelines |  | 3, 6, 9, 18, 33 | 3, 4 | Health system factors |
| 11 | Lack of adequate service user records |  | 3, 5 | 3, 4 | Health system factors |
| 12 | Limited theatre time |  | 2, 6, 7, 8, 14, 33 | 3 | Health system factors |
| 13 | Lack of supervision for junior service providers |  | 9, 17, 20 | 3, 4 | Health system factors |
| 14 | Cancellation of appointments and procedures | Cancellations from both the service provider level and the service user. | 3, 6, 7, 8, 9, 12, 20, 21, 22, 23, 24, 26, 27, 30, 33 | 2, 3, 4 | Health system factors |
| 15 | Lack of surgical health education | Educational material about surgical conditions unavailable or inaccessible to service users | 2, 17, 25 | 1, 4 | Health system factors |
| 16 | Lack of decentralised services |  | 1, 2, 3, 4, 6, 9, 18 | 1, 2, 3, 4 | Health system factors |
| 17 | Lack of service provider's knowledge, training and experience |  | 2, 6, 9, 13, 15, 27 | 3, 4 | Health system factors |
| 18 | Lack of home based services | No provision of health services, supported self-care and health education at home | 2, 3, 4, 10, 16, 33 | 4 | Health system factors |
| 19 | Limited surgical outreach | Lack of surgical care at peripheral healthcare facilities | 2, 6, 29 | 3, 4 | Health system factors |
| 20 | Previous bad experiences |  | 9, 13, 14, 21, 22, 23, 24, 27, 28, 31, 32, 34 | 1 | Individual factors - Service user |
| 21 | Wariness of healthcare system | Lack of trust in the healthcare system due to its reputation and previous bad experiences | 3, 6, 7, 9, 14, 20, 22, 23, 24, 27, 32 | 1 | Individual factors - Service user |
| 22 | Fear of the individual consequences of surgical care | Individual consequences such as loss of income and complications /disability after surgery | 14, 20, 21, 23, 24, 25, 26, 28, 31 | 1 | Individual factors - Service user |
| 23 | Procrastination |  | 9, 14, 20, 21, 22, 24, 25, 26, 27, 28, 31 | 1, 4 | Individual factors - Service user |
| 24 | Religion / preference for alternative practitioners |  | 3, 4, 6, 9, 14, 20, 21, 22, 23, 25, 27, 28, 31, 32, 33, 34 | 1, 4 | Individual factors - Service user |
| 25 | Lack of service user's surgical healthcare knowledge | Service users are unable or unwilling to assimilate available health education material | 15, 22, 23, 24, 28, 31, 34 | 1, 4 | Individual factors - Service user |
| 26 | Family responsibilities | Includes caring for family members and being the breadwinner of the family | 1, 14, 22, 23, 28 | 1, 2, 4 | Individual factors - Service user |
| 27 | Experienced lack of staff empathy |  | 3, 14, 17, 20, 21, 23, 24, 32, 34 | 1, 3, 4 | Individual factors – service user |
| 28 | Lack of social support | Includes emotional support, help with family responsibilities and physical care for the service user in the pre and postoperative period | 1, 20, 22, 23, 24, 25, 26, 31 | 1, 2, 4 | Non-healthcare infrastructural factors |
| 29 | Poor road infrastructure |  | 1, 19 | 2, 4 | Non-healthcare infrastructural factors |
| 30 | Difficulty with navigating the facility | Service users have difficulty finding their specific points of care once they've reached the healthcare facility | 14, 34 | 3, 4 | Non-healthcare infrastructural factors |
| 31 | Stigma of disease | Disgrace and shame associated with surgical conditions | 20, 22, 23, 24, 25, 28, 32 | 1,4 | Societal factors |
| 32 | Systemic inequities | Prejudice ingrained in all participants of the healthcare system influencing access to and provision of care | 3, 7, 9, 20, 21, 24, 27, 31 | 1,3, 4 | Societal factors |
| 33 | Large burden of disease |  | 3, 6, 7, 9, 10, 12, 14, 18, 24 | 3, 4 | Societal factors |
| 34 | Language barrier |  | 5, 9, 20, 24, 25, 27, 30 | 1, 3, 4 | Societal factors |

*Delay 1: seeking care, Delay 2: reaching care, Delay 3: receiving care, Delay 4: remaining in care.

**
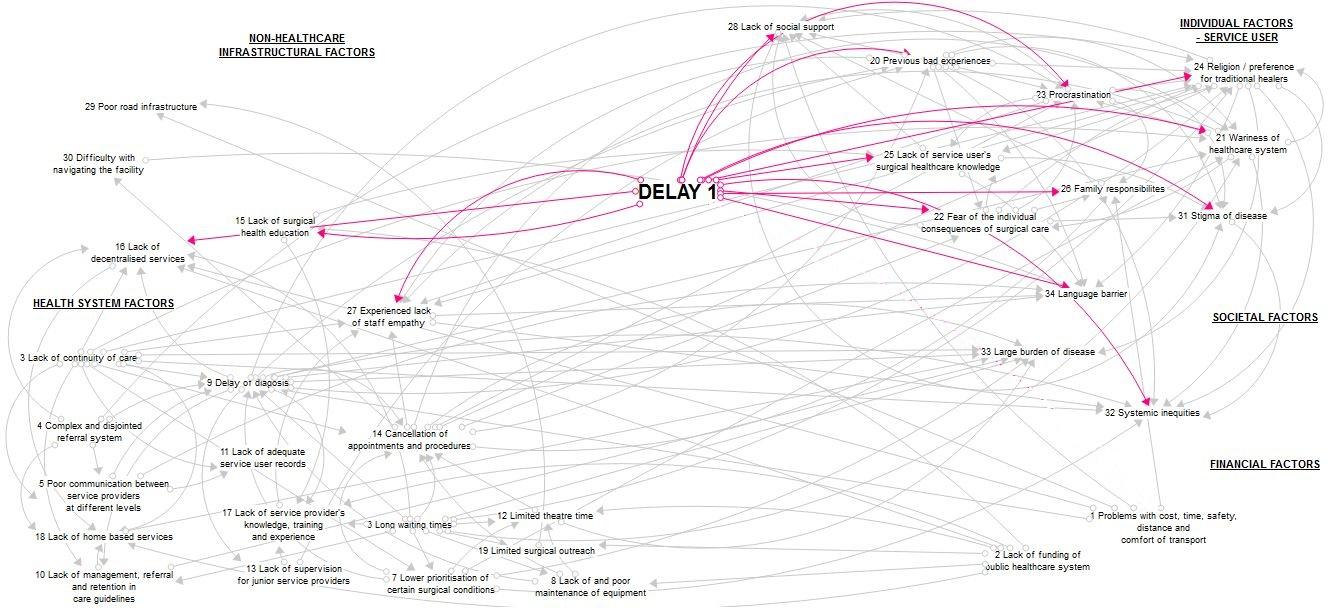
**

Supplementary Figure 1: Map showing barriers, their interconnection and their causative relationship with Delay 1.


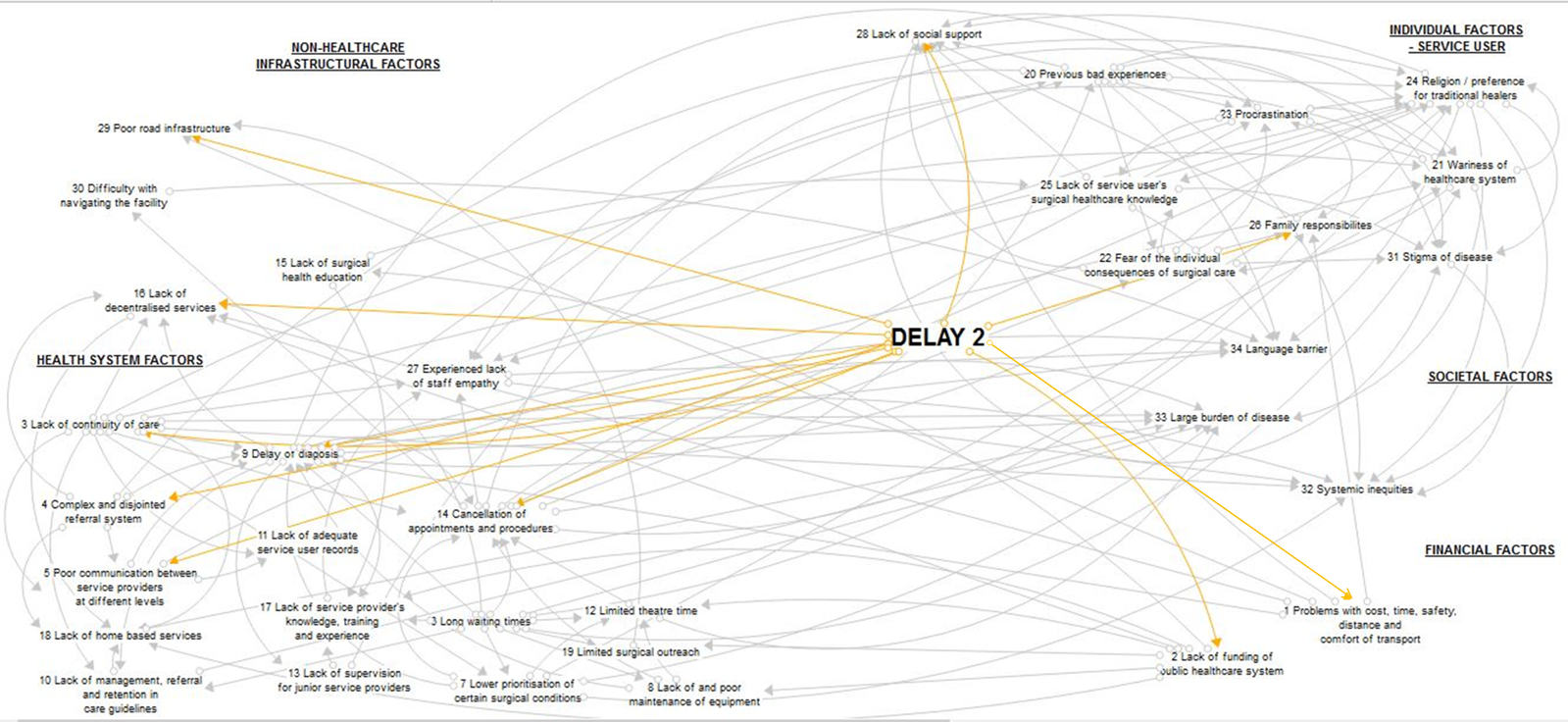


Supplementary Figure 2: Map showing barriers, their interconnection and their causative relationship with Delay 2.

**
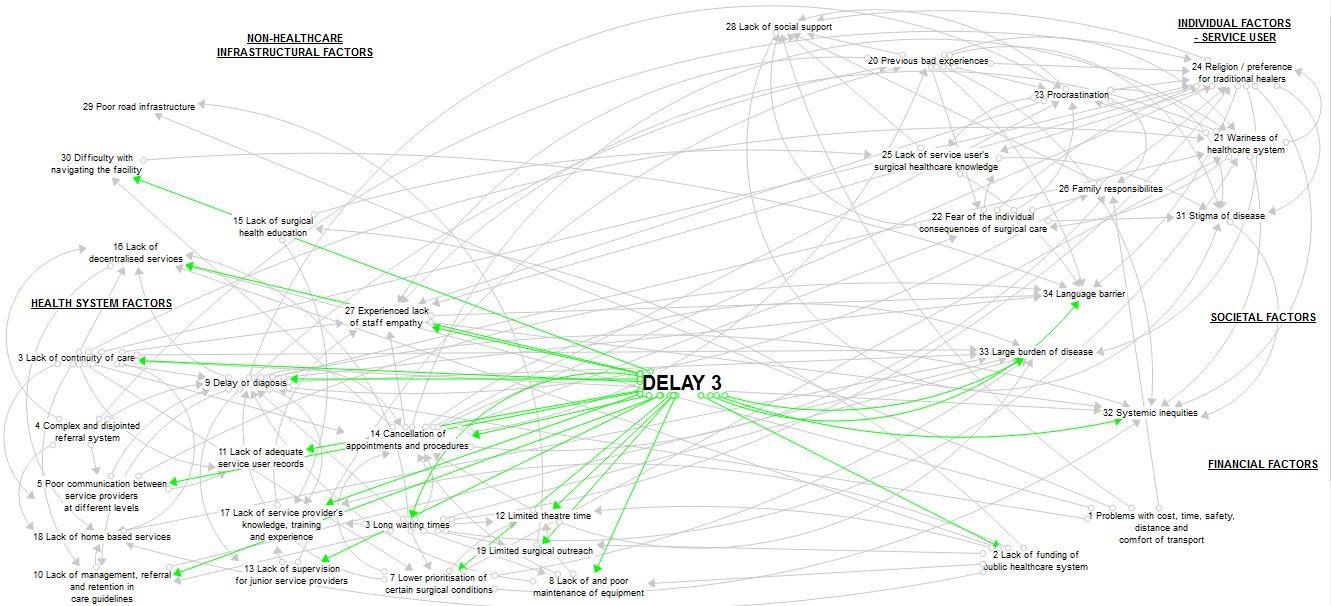
**

Supplementary Figure 3: Map showing barriers, their interconnection and their causative relationship with Delay 3.


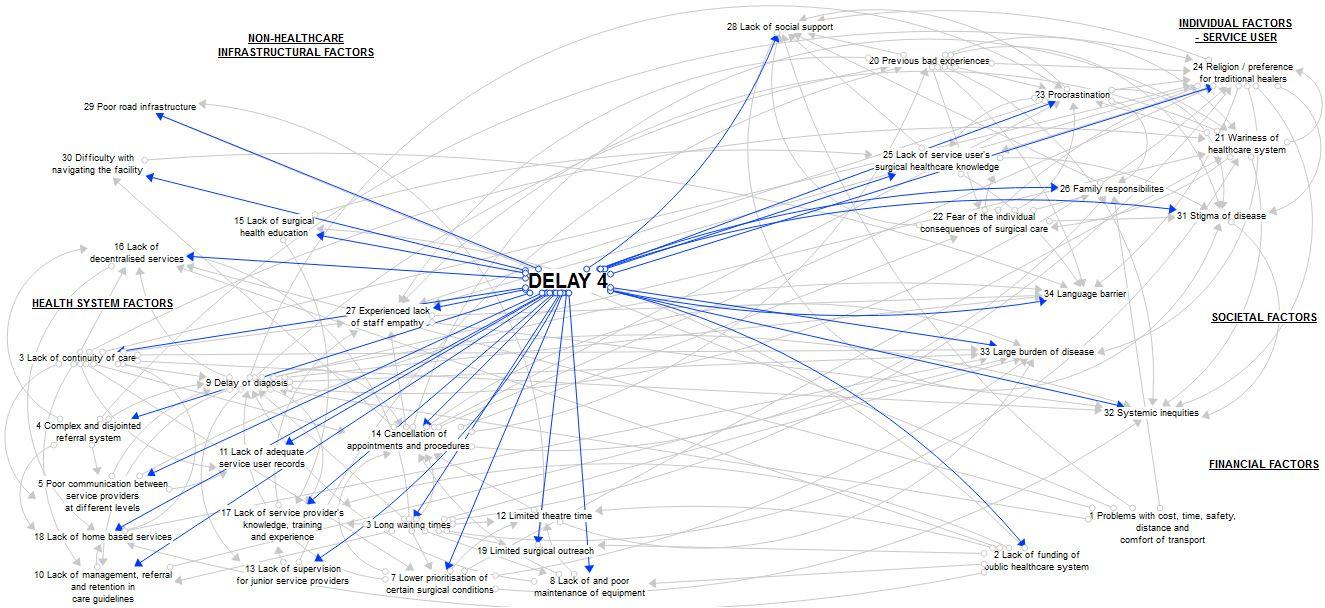


Supplementary Figure 4. Map showing barriers, their interconnection and their causative relationship with Delay 4.
